# Generalized Maculopapular Eruption Induced by Minocycline: A Case Report

**DOI:** 10.7759/cureus.114424

**Published:** 2026-08-12

**Authors:** Shruti Brahmbhatt, Arpit M Shah

**Affiliations:** 1 Pharmacology, Smt. Bhikhiben Kanjibhai Shah Medical Institute and Research Centre, Sumandeep Vidyapeeth, Vadodara, IND

**Keywords:** adverse-drug reaction, maculopapular rash, minocycline, multidrug-resistant klebsiella pneumoniae, ventilator-associated pneumonia

## Abstract

Ventilator-associated pneumonia is a frequent cause of infection that is responsible for high morbidity and mortality, particularly when caused by multidrug-resistant gram-negative organisms. Minocycline is useful for its broad antimicrobial spectrum. Minocycline may cause gastrointestinal upset, dizziness, pruritus, photosensitivity, drug-induced lupus, intracranial hypertension, and drug reaction with eosinophilia and systemic symptoms (DRESS) syndrome, including rare adverse reactions such as cutaneous manifestations. Here, we report an uncommon case of a 51-year-old male patient admitted to the medical intensive care unit (MICU) due to ventilator-associated pneumonia caused by multidrug-resistant *Klebsiella pneumoniae*. The patient developed a generalized maculopapular rash within 12 hours of receiving a single dose of 200 mg of minocycline through the intravenous route. The medication was stopped the next day. The patient received supportive treatment with levocetirizine, and the rash disappeared completely within 48 hours. Causality assessment using the Naranjo Adverse Drug Reaction Probability Scale yielded a score of 6, and the World Health Organization-Uppsala Monitoring Centre (WHO-UMC) scale categorized the event as “probable.” This case underlines the need for close monitoring of patients for cutaneous adverse drug reactions during minocycline therapy. Early identification and immediate withdrawal of the drug are essential for rapid recovery.

## Introduction

Minocycline is a semisynthetic member of the tetracycline class that inhibits bacterial growth. Its broad-spectrum classification stems from its ability to target a wide variety of pathogens, encompassing gram-positive and gram-negative organisms [1].

The clinical applications of minocycline include treating various dermatological, respiratory, and genitourinary infections. It serves as a therapeutic option for diseases caused by gonorrhea, syphilis, chlamydia, and *Mycoplasma*. As an old antibiotic, minocycline is being reintroduced in a new and vital way for the intensive care of infections caused by multidrug-resistant pathogens [2].

One of the most common and fatal lung infections in the intensive care unit (ICU) is ventilator-associated pneumonia (VAP), which develops as a complication associated with mechanical ventilation. It negatively affects the recovery of critically ill patients, and the shortage of effective new drugs to treat multidrug-resistant strains continues to be a complex obstacle [3].

Adverse effects from systemic minocycline can affect the nervous, gastrointestinal, musculoskeletal, respiratory, cutaneous, and genitourinary systems. Common cutaneous adverse effects include urticaria, pruritus, and erythematous rash, while the rare cutaneous adverse effects are angioedema, alopecia, hyperpigmentation, photosensitivity, cutaneous vasculitis, drug reaction with eosinophilia and systemic symptoms (DRESS) syndrome, and maculopapular rash [4].

The primary objective of this case report is to highlight an infrequently reported, hyper-acute presentation of a generalized maculopapular drug eruption occurring 12 hours after the administration of an intravenous (IV) minocycline loading dose. While minocycline-induced cutaneous reactions are well-documented, most maculopapular eruptions and severe reactions such as DRESS syndrome present after delayed latency periods ranging from several days to two to six weeks. Rapid-onset eruptions occurring within hours of initiation are infrequently reported, particularly with IV loading doses. By contrasting this rapid reaction with the typical delayed-onset cutaneous manifestations of the drug, this report aims to emphasize the necessity for immediate and vigilant dermatological monitoring in critical care patients undergoing targeted therapy for multidrug-resistant infections.

This article was previously presented as a poster at the 45th Annual National Conference of the National Medicos Organisation (NMOCON 2026) held on March 21-22, 2026, at Medical College Baroda, Vadodara, Gujarat, India.

## Case presentation

Patient history

A 51-year-old male patient was admitted following a traumatic brain injury that required emergency craniotomy and evacuation of a right fronto-temporo-parietal subdural hemorrhage. His post-operative course was complicated by an episode of seizure, eventually requiring mechanical ventilation and a tracheostomy.

Throughout this period, the patient was maintained on a stable treatment protocol including sodium valproate tablet (500 mg), lacosamide tablet (100 mg), and brivaracetam tablet (100 mg) twice daily. Antimicrobial support consisted of nebulized colistin (1 million IU) three times daily and cefoperazone/sulbactam (3 g) twice daily through the IV route, alongside supportive care and neuro-rehabilitation with carvedilol tablet (3.125 mg), pantoprazole tablet (40 mg), and a citicoline/piracetam tablet (500/800 mg) combination.

Upon the clinical manifestation of VAP caused by multidrug-resistant *Klebsiella pneumoniae*, 200 mg of minocycline was initiated through the IV route following microbiological sensitivity results. Within 12 hours of the first dose of minocycline, the patient developed a generalized, blanchable, erythematous maculopapular rash on the abdomen and bilateral thighs.

Minocycline was immediately discontinued, leading to the disappearance of the rash within 48 hours. The close temporal relationship between drug administration and the onset of the rash, combined with a positive de-challenge upon drug withdrawal, led to a clinical diagnosis of a probable minocycline-induced cutaneous adverse drug reaction (ADR).

Physical examination

On physical examination, the patient was afebrile with stable vital signs, maintaining a heart rate of 78 beats per minute and a blood pressure of 120/80 mmHg on minimal ventilatory support. Cutaneous examination revealed a generalized eruption consisting of multiple, discrete erythematous papules overlying a background of blanchable erythema. The rash was widespread, involving approximately 15% to 20% of the total body surface area (BSA), with dense involvement prominently documented across bilateral anterior thighs (Figure 1) and the abdomen (Figure 2).

**Figure 1:**
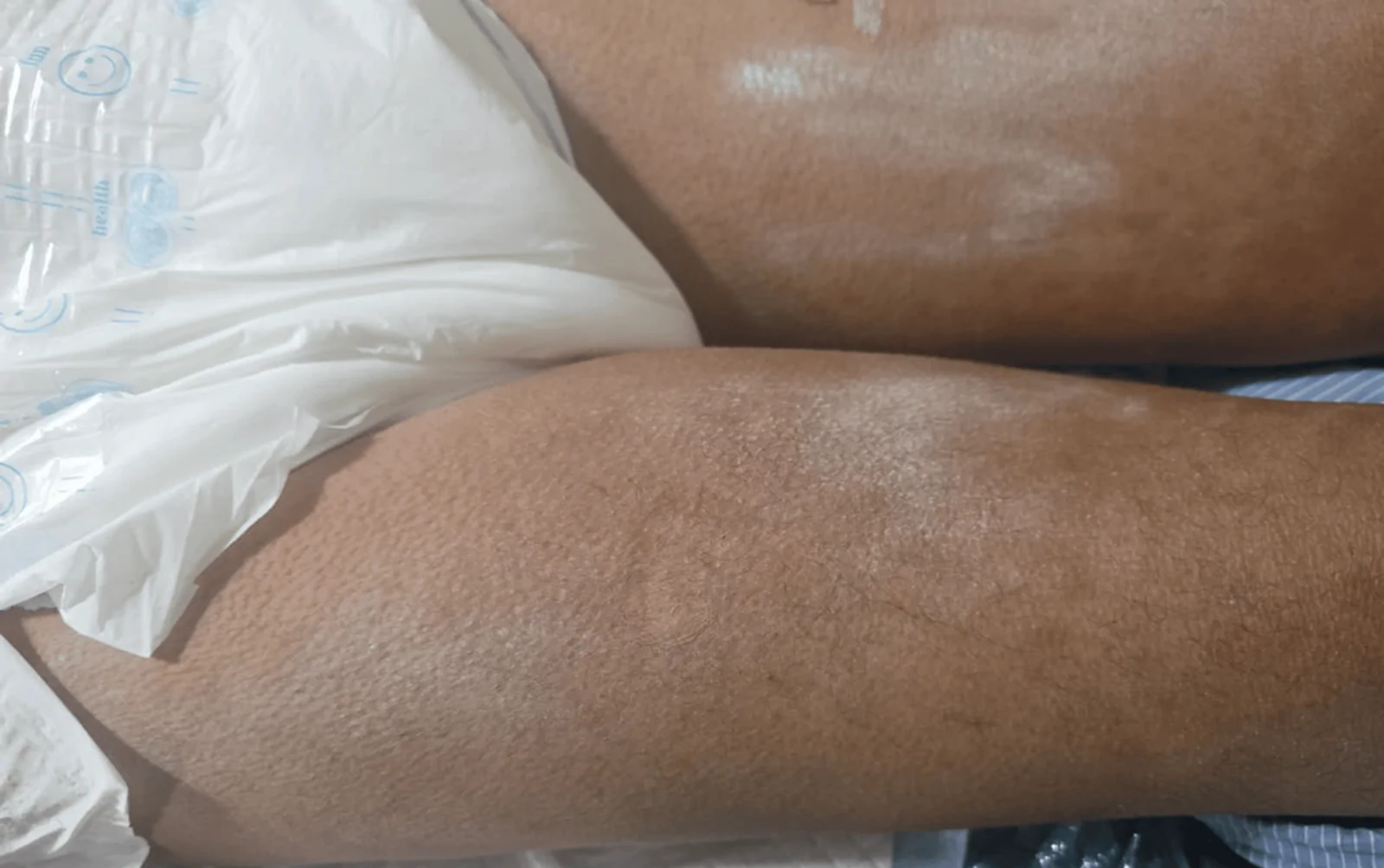
Erythematous papules with underlying blanchable erythema on bilateral thighs, taken at the onset of the eruption.

**Figure 2:**
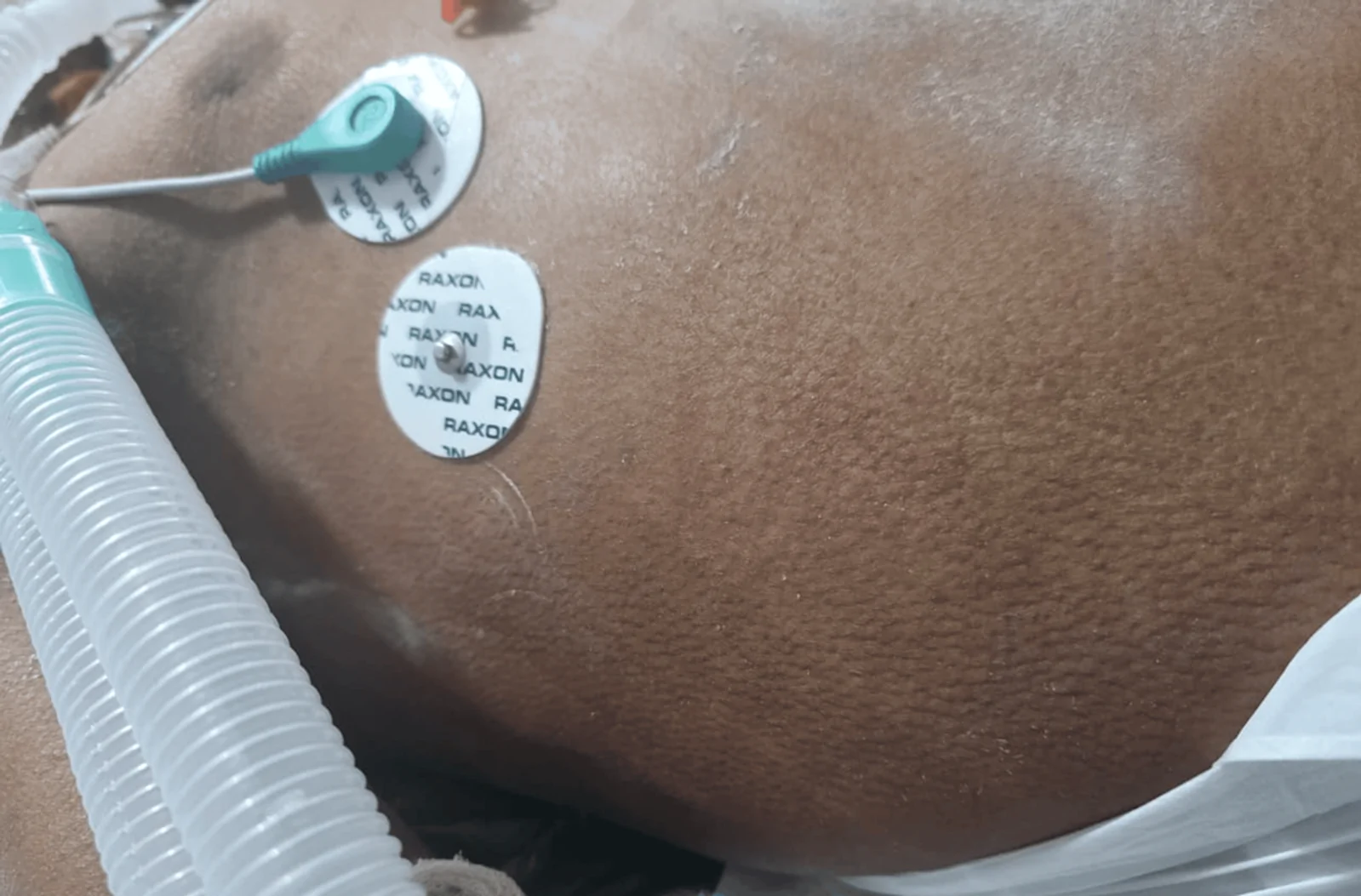
Erythematous papules with underlying blanchable erythema on the abdomen, taken at the onset of the eruption.

Importantly, there was no clinical evidence of facial edema, palpable lymphadenopathy, or tenderness upon palpation of the affected skin. Mucosal examination revealed completely clear oral, ocular, and genital mucosae, with no signs of congestion or ulceration. Furthermore, the skin surface showed no evidence of vesiculation, bullae formation, target lesions, or desquamation. Systemic examinations, including cardiovascular, abdominal, and central nervous systems, were unremarkable and unchanged from the patient's baseline clinical status.

Past history

The patient had a long-standing history of hypertension and a social history of chronic alcohol abuse extending over 15 to 20 years. Crucially, there was no prior history of drug allergies or atopy. The patient had recently undergone an emergency right fronto-temporo-parietal craniotomy for the evacuation of an acute subdural hemorrhage, necessitating subsequent transfer to the ICU for mechanical ventilation and post-operative monitoring. The patient had no documented inpatient or outpatient history of prior exposure to tetracyclines or minocycline; however, unrecorded previous outpatient sensitization cannot be completely excluded and may account for the rapid onset of the eruption.

Laboratory investigation

The laboratory results are presented in Tables 1-3.

**Table 1:** Serological panel demonstrating elevated C-reactive protein secondary to active ventilator-associated pneumonia, alongside non-reactive viral screens. HCV: hepatitis C virus

Test name	Result	Biological reference interval
C-reactive protein method: nephelometry	91.15 mg/L	0-6 mg/L
Anti-HCV antibody method: rapid immunochromatography	Negative	-
HBsAg method: rapid immunochromatography	Negative	-
HIV (1 & 2) method: rapid immunoconcentration	Non-reactive	-

**Table 2:** Hematological profile showing leukocytosis and a normal differential leukocyte count to explicitly exclude eosinophilia and hematological disorders.

Test name	Result	Biological reference interval
Hemoglobin	13.2 g/dL	13-17 g/dL
Total WBC count	13,670/cumm	4,000-10,000/cumm
Total RBC count	4.7 mill/cumm	4.5-5.5 mill/cumm
Neutrophil	71%	40%-80%
Lymphocyte	20%	20%-40%
Eosinophil	4%	1%-6%
Monocyte	5%	2%-10%
Basophil	0%	0%-2%
Platelet count	1.70 Lac/cmm	1.5-4.0 Lac/cmm

**Table 3:** Biochemical profile demonstrating unremarkable renal and hepatic parameters with intact electrolyte balance to exclude systemic organ failure. SGOT: serum glutamic oxaloacetic transaminase; SGPT: serum glutamic pyruvate transaminase

Test name	Result	Biological reference interval
Renal function test		
Blood urea	40 mg/dL	15-40 mg/dL
S. creatinine	1.1 mg/dL	0.7-1.3 mg/dL
Liver function test		
S. bilirubin total	1.0 mg/dL	0.2-1.0 mg/dL
S. SGPT	28 U/L	0-45 U/L
S. SGOT	32 U/L	0-35 U/L
S. sodium	136 mmol/L	136-145 mmol/L
S. potassium	4.1 mmol/L	3.5-5.1 mmol/L
S. chloride	100	98-107

Differential diagnosis

Identifying the cause of the maculopapular rash required a systematic exclusion of infectious and pharmacological variables. Common blood-borne viral pathogens were excluded via non-reactive serology for HIV, HBsAg, and hepatitis C virus (HCV). Although specific serological testing for acute exanthematous viruses was not performed, an infectious viral etiology was deemed clinically unlikely given the absence of fever, lack of mucosal or respiratory prodromal symptoms, and the rapid, complete resolution of the eruption within 48 hours following minocycline discontinuation.

The primary challenge involved the patient's maintenance of polypharmacy, specifically the antiepileptics sodium valproate, lacosamide, and brivaracetam. These were excluded due to long-term tolerance and stable dosing during the post-operative period. The diagnosis was considered probable by the strict 12-hour temporal correlation between the first minocycline dose and the eruption. This was followed by a definitive de-challenge; the rash resolved within 48 hours of drug discontinuation.

Treatment

Following the clinical manifestation of the maculopapular rash after a single dose of 200 mg of minocycline through the IV route, minocycline was immediately discontinued. Management was primarily supportive, involving the administration of oral levocetirizine (5 mg) twice daily for symptomatic relief. Topical calamine lotion was applied to the affected areas twice daily. Systemic corticosteroids were not initiated as the eruption was mild, localized, and devoid of mucosal or organ involvement. Avoiding unnecessary systemic corticosteroid exposure was prioritized in this critically ill patient with active multidrug-resistant VAP.

Outcome and follow-up

A rapid clinical response was observed post-discontinuation of minocycline; the rash disappeared significantly within 24 hours and reached complete resolution by 48 hours. The Naranjo Adverse Drug Reaction Probability Scale score [5] for minocycline was 6, and the World Health Organization-Uppsala Monitoring Centre (WHO-UMC) Causality Assessment Scale [6] also categorized the event as "probable." The event met WHO-UMC “probable” criteria due to a plausible temporal sequence relative to drug intake, a clear clinical recovery following drug withdrawal (de-challenge), and the improbability of attributing the eruption to concurrent medications following individual causality assessment. To firmly establish causality within this polypharmacy setting, a comprehensive differential assessment was performed for all concurrent medications. The concurrent antimicrobials, IV cefoperazone/sulbactam, and nebulized colistin, as well as the gastrointestinal prophylaxis with pantoprazole, were graded as "doubtful" (Naranjo score: 1) and "unlikely" under the WHO-UMC criteria [6], as they were continued without interruption while the rash completely cleared, representing a negative de-challenge. Similarly, the stable post-surgical maintenance regimen-comprising sodium valproate, lacosamide, brivaracetam, carvedilol, and the citicoline/piracetam combination-demonstrated no chronological correlation to the acute eruption, consistently yielding a Naranjo score [5] of 0 (doubtful) and a WHO-UMC classification [6] of unlikely. Following minocycline discontinuation, the underlying VAP was successfully managed with the continued regimen of IV cefoperazone/sulbactam and nebulized colistin, leading to complete resolution. The patient remained stable throughout the hospital stay, eventually reaching a full recovery with no residual skin changes due to the isolated nature of this hyper-acute cutaneous reaction.

Causality assessment using the Naranjo Adverse Drug Reaction Probability Scale yielded a total score of 6, categorizing the reaction as “probable” [5]. This score was calculated based on positive findings for previous conclusive reports (+1), appearance of the eruption post-drug administration (+2), rapid recovery upon de-challenge (+1), and the absence of alternative non-pharmacological or infectious etiologies (+2), with no re-challenge, placebo administration, or toxic concentration monitoring performed (0).

## Discussion

VAP is a serious and common infection of the lung tissue that develops because of mechanical ventilation. It represents the most important factor in determining the recovery of the patient [7].

VAP is defined as an infection of the lungs that develops after 48 hours of endotracheal intubation. It is associated with a 13% mortality rate. Because of this high clinical burden, potent antibiotic strategies are required, involving broad-spectrum drugs to treat multidrug-resistant organisms [8].

The clinical basis for using minocycline is supported by its high lipophilic property, which facilitates excellent penetration into lung parenchyma and bronchial secretions, reaching concentrations up to four times higher than those found in plasma [9]. In cases involving multidrug-resistant *K. pneumoniae*, minocycline serves as an important medicine due to its ability to bypass certain efflux pump-mediated resistance mechanisms [10].

Minocycline exerts its effects by attaching to the 30S ribosomal subunit of bacteria, which halts the production of essential proteins. This FDA-authorized semisynthetic tetracycline is known for its broad-spectrum properties [11].

Most documented cases of minocycline show that minocycline is usually associated with delayed, serious reactions like DRESS syndrome, which often takes two to six weeks to develop and can damage internal organs [12]. While some cases of urticaria have been reported within 15 days of initiation of minocycline, our case is uncommon due to its hyper-acute presentation within just 12 hours of a loading dose [13]. This clinical pattern suggests a presumed Type IV hypersensitivity response.

An ADR is defined by the WHO as any response to a drug that is noxious and unintended, occurring at doses normally used in humans for prophylaxis, diagnosis, or therapy of disease [14]. Minocycline-related skin reactions are uncommon but clinically important ADRs observed in critical care while treating multidrug-resistant infection pathogens. Minocycline's high lipophilicity enhances its tissue distribution and epidermal penetration, which may facilitate localized dermal exposure during hypersensitivity responses [15]. In the absence of systemic organ failure or hematological abnormalities, the eruption was clinically consistent with a localized drug-induced cutaneous reaction rather than a metabolic or primary infectious process [16].

## Conclusions

This case illustrates an uncommon, hyper-acute presentation of an isolated cutaneous reaction clinically suspected to represent a Type IV hypersensitivity response precipitated by an IV minocycline loading dose. While clinical literature extensively links minocycline to delayed-onset systemic hypersensitivity syndromes-most notably DRESS syndrome, which presents after a distinct two-to-six-week latency period-this presentation demonstrates that severe immunologic skin reactions can erupt within a mere 12 hours of initial exposure. The complete absence of systemic organ involvement, mucosal lesions, and hematological abnormalities underscores that rapid-onset, drug-induced cutaneous reaction can remain localized to the dermis. This underscores a unique diagnostic challenge in intensive care environments where complex polypharmacy often obscures the identity of the true offending agent.

Ultimately, this presentation serves as a critical clinical reminder that the established pharmacokinetic and adverse-effect profiles of old, reintroduced antibiotics should not delay the recognition of immediate hypersensitivity events. Early detection remains a powerful diagnostic and therapeutic tool; as observed here, immediate cessation of minocycline combined with standard supportive measures can lead to a complete clinical resolution within 48 hours without any residual skin changes. Clinicians must maintain a high index of suspicion and practice vigilant dermatological monitoring during the first 24 hours of initiating minocycline therapy to ensure patient safety and optimize treatment outcomes in critically ill patients fighting multidrug-resistant infections.
